# Feasibility of Sodium and Amide Proton Transfer-Weighted Magnetic Resonance Imaging Methods in Mild Steatotic Liver Disease

**DOI:** 10.3390/tomography11080089

**Published:** 2025-08-06

**Authors:** Diana M. Lindquist, Mary Kate Manhard, Joel Levoy, Jonathan R. Dillman

**Affiliations:** Imaging Research Center, Department of Radiology, Cincinnati Children’s Hospital Medical Center, University of Cincinnati College of Medicine, 3333 Burnet Ave, Cincinnati, OH 45229-3026, USA; marykate.manhard@cchmc.org (M.K.M.); joel.levoy@cchmc.org (J.L.); jonathan.dillman@cchmc.org (J.R.D.)

**Keywords:** sodium MRI, amide proton transfer-weighted MRI, liver fibrosis, steatotic liver disease

## Abstract

**Background/Objectives**: Fat and inflammation confound current magnetic resonance imaging (MRI) methods for assessing fibrosis in liver disease. Sodium or amide proton transfer-weighted MRI methods may be more specific for assessing liver fibrosis. The purpose of this study was to determine the feasibility of sodium and amide proton transfer-weighted MRI in individuals with liver disease and to determine if either method correlated with clinical markers of fibrosis. **Methods**: T_1_ and T_2_ relaxation maps, proton density fat fraction maps, liver shear stiffness maps, amide proton transfer-weighted (APTw) images, and sodium images were acquired at 3T. Image data were extracted from regions of interest placed in the liver. ANOVA tests were run with disease status, age, and body mass index as independent factors; significance was set to *p* < 0.05. Post-hoc t-tests were run when the ANOVA showed significance. **Results**: A total of 36 participants were enrolled, 34 of whom were included in the final APTw analysis and 24 in the sodium analysis. Estimated liver tissue sodium concentration differentiated participants with liver disease from those without, whereas amide proton transfer-weighted MRI did not. Estimated liver tissue sodium concentration negatively correlated with the Fibrosis-4 score, but amide proton transfer-weighted MRI did not correlate with any clinical marker of disease. **Conclusions**: Amide proton-weighted imaging was not different between groups. Estimated liver tissue sodium concentrations did differ between groups but did not provide additional information over conventional methods.

## 1. Introduction

Liver diseases are characterized by fibrosis, inflammation, and fat deposition. Fibrosis may be the most important predictor of mortality in chronic liver disease [1,2] and is the target of several emerging therapeutics [3,4]. Liver biopsy is currently the standard of care to quantify fibrosis, but it is limited by sampling error and is not suitable for long-term follow up. A non-invasive method of quantifying fibrosis would facilitate the testing of new therapeutics and enable the long-term monitoring of patients with liver disease.

Fibrosis occurs when excess collagen and extracellular matrix proteins are deposited due to tissue injury. While magnetic resonance imaging (MRI) contrast agents targeting collagen have been described [5], they are not in clinical use and are, furthermore, gadolinium-based, which can cause nephrogenic systemic fibrosis [6,7] and may be neurotoxic [8,9]. Collagen is composed of amino acids, which form amide bonds as the collagen chain lengthens [10]. Increased levels of hepatic extracellular matrix protein also result in increased levels of proteoglycans [11]. Proteoglycans contain negatively charged glycosaminoglycan side chains, which attract positive cations such as sodium. Thus, imaging methods that probe either collagen levels via amide protons or proteoglycan concentration via sodium increases may be useful for quantifying fibrosis.

Amide proton transfer-weighted (APTw) imaging [12,13,14] is a technique sensitive to the presence of amide protons and, therefore, is expected to correlate with the amount of collagen present. For APTw imaging, a selective radiofrequency saturation pulse is applied at 3.5 ppm from water to saturate exchangeable amide protons, which then results in a decrease in the subsequently measured water signal. However, the decreased water signal can result not just from exchangeable amide protons but also from magnetization transfer due to exchangeable protons on macromolecules. To control for this, images are acquired with the offset of the radiofrequency pulse mirrored around water at −3.5 ppm. The APTw image can then be calculated from the magnetization transfer ratio (MTR) images at offsets of ±3.5 ppm as follows:

(1) $${MTR}_{\pm 3.5\;ppm}=1-\frac{{SI}_{\pm 3.5\;ppm}}{{SI}_{ref}}$$

(2) $$APTw={MTR}_{-3.5\;ppm}-{MTR}_{+3.5\;ppm}$$

where *SI* is the signal intensity at the indicated offsets and *SI_ref_* is the signal intensity of a reference image collected without a saturation pulse. Fat, since it resonates at −3.5 ppm from water, can artificially decrease the calculated APTw signal [15], but fat suppression techniques can mitigate this interference [16].

APTw imaging has been applied to the detection and staging of brain cancer [12,13], ischemic stroke [14,17], neurological diseases [18], and breast and prostate cancer [19]. Although it has been used to evaluate liver lesions [16] and the effects of fasting [20], we were unable to find literature on its application to the detection of human liver fibrosis. However, in animal models of liver fibrosis, Chen showed that APTw imaging was sensitive to acute CCl_4_ intoxication in rat liver [21], and we found that APTw imaging correlates with histological markers of fibrosis in mice treated with CCl_4_ for 16 weeks [22].

Similarly, sodium MRI has been used to study cartilage, neuromuscular disorders, neurological disorders, and cancers, as reviewed by Gast et al. [23]. Among these, sodium MRI has been shown to correlate with cartilage proteoglycan content [24]. We have previously shown that sodium MRI correlates with fibrosis as assessed by Sirius Red staining in a mouse model [22].

The purpose of this study was to acquire sodium and APTw images from the livers of participants with and without mild steatotic liver disease to determine if sodium levels or APTw signal intensity differed between groups. A secondary aim was to correlate these measurements with clinical measures of liver disease. We hypothesized that the sodium and APTw signals would be increased in liver disease and correlate with histological measures of fibrosis.

## 2. Materials and Methods

### 2.1. Participants

This prospective, cross-sectional study was approved by the local Institutional Review Board and performed in accordance with the Declaration of Helsinki and its amendments. Written informed consent or assent was obtained from all participants or their parents/guardians, as appropriate. The APTw study and sodium study were conducted under approved, but separate, protocols.

Participants were recruited and examined between November 2022 and March 2023 from either our institutional Steatohepatitis Center (patients with biopsy-proven liver disease) or via department-wide emails to Department of Radiology staff (healthy participants with no known liver disease). All participants were between 11 and 25 years old. Patients were included in our study if a biopsy was performed within the preceding 12 months. Exclusion criteria included contraindication to MRI and known liver disease in healthy control participants. Healthy participants who were found to have a proton density fat fraction (PDFF) >6% at research MRI were excluded from the study. As this was a preliminary study, we planned to recruit 20 participants in each group for the APTw study; assuming similar results as for our murine study, we expected this to provide greater than 95% power to distinguish the groups. For the sodium study, only 25 participants could be enrolled due to funding limits.

### 2.2. APTw Imaging Optimization

Collagen phantoms consisting of 5, 10, and 15% NeoCell (NeoCell, Ronkonkoma, NY, USA) collagen in a copper-doped saline solution were used to optimize the saturation pulse power and duration within the constraints imposed by the available imaging time and hardware and specific-absorption rate limits on the radiofrequency (RF) pulse power. The number, duration, and strength of the saturation pulses were varied to find parameters that mimicked the phantom results from the murine study as closely as possible.

### 2.3. Image Acquisition

The research MRI scan was performed on a 3-Tesla MRI scanner (Ingenia; Philips Healthcare, Best, The Netherlands). Proton imaging included T_1_ and T_2_ relaxation time mapping, PDFF mapping (using an mDixon QUANT^TM^ sequence), MR elastography, and APTw imaging using the parameters determined from the phantoms and offsets of ±3.5 ppm and 23.5 ppm from water. All proton scans were completed using a transmit body coil and a 32-channel anterior/posterior receive phased array coil with body coil transmit (Philips Healthcare). A single 10-mm thick slice through the level of the porta hepatis using a 500 mm × 500 mm field-of-view was acquired for all data.

After the proton study, the subjects were removed from the scanner and then repositioned with a sodium surface coil (PulseTeq, Ltd., Surrey, UK) placed over the liver. A proton localizer was acquired to confirm coil placement, after which a sodium image was acquired using a 500 mm × 500 mm FOV from a 20 mm thick slice through the porta hepatis. No sodium B_1_ map was acquired for subject scans due to time constraints. Reference vials containing 50, 75, 100, and 154 mM NaCl were attached to the exterior of the coil to use in calculating the apparent tissue sodium concentration. The low spatial resolution of the sodium scans was required to achieve a short echo time and an acceptable signal-to-noise ratio in the resulting image.

To determine relaxation times for the reference vials, which were needed to estimate sodium concentration, a single reference scan was performed using a phantom with 50 mM NaCl in place of the subject to load the coil. The phantom was placed at the average distance of the liver from the coil for the healthy subjects. A multiple gradient echo version of the imaging sequence was used with 5 echo times from 2.3 to 15.2 ms to estimate T_2_*. T_1_ relaxation was calculated from a pair of images acquired at flip angles of 15 and 30 degrees.

Additional sequence details are provided in Table 1.

T_1_, fat fraction, and liver stiffness maps were reconstructed on the scanner using vendor-provided software. T_2_, MTR, and APTw maps were calculated using in-house software (MATLAB 24.2; The MathWorks, Inc., Natick, MA, USA). The signal intensity at the 23.5 ppm offset was used as the reference for the APTw calculations; acquiring the reference as part of the image series controlled for receiver gain and other possibly variable scan settings. T_2_ maps and the T_2_* values for the sodium reference vials were found from fitting the images at multiple echo times to a monoexponential decay. MTR and APTw maps were calculated using Equations (1) and (2) above. T_1_ for the sodium reference vials was calculated using the multiple flip angle method [25].

### 2.4. Histology

Histology scores were extracted from electronic health records. The scores were assigned according to the NASH CRN criteria [26] by a clinical pathologist. Fibrosis scores (0, 1, 1a, 1b, 1c, 2–4) were assigned based on the appearance, location, and extent of fibrosis observed in biopsy samples. The alphanumeric scores were converted to numeric values with 1a = 1.25, 1b = 1.5, 1c = 1.75 for statistical analysis. Similarly, the nonalcoholic fatty liver disease activity score [NAS, a score of 0–8, which is the sum of histologic scores for steatosis (0–3), lobular inflammation (0–3), and hepatocyte ballooning (0–2) [27] was assigned based on the appearance of the biopsy sample. Values for alanine transaminase (ALT) levels, aspartate aminotransferase (AST) levels, and platelet count were extracted from the electronic health records of the participants with liver disease. The Fibrosis-4 (Fib4) score [28] is a biomarker that is commonly used in lieu of biopsy to evaluate the risk of liver fibrosis [29]. The Fib4 was calculated from ALT, AST, and the platelet count according to [28]

(3) $$Fib4=\frac{\left(age*AST\right)}{(platelet\;count*\sqrt{ALT)}}$$

Biopsies and blood work were completed within the 12 months prior to the MRI research study. All participants had height and weight recorded at the time of the MRI exam to calculate body mass index (BMI).

### 2.5. Extraction of Parametric MRI Measures

For proton data, elliptical or circular regions-of-interest (ROIs), approximately 1 cm in area, were placed in the anterior, central, and posterior regions of the right liver lobe. These regions were used to extract the parametric measures using ImageJ v1.52s, National Institutes of Health, MD, USA [30]. The average value of each measurement was used for subsequent statistical analysis.

For sodium data, circular ROIs were placed on the reference vials. The proton localizer image was used to draw one ROI in the liver. These ROIs were transferred to the sodium image to extract sodium signal intensity. To account for the signal drop off associated with the use of a surface coil, the measured sodium signal was corrected for the distance from the coil by multiplying by the distance to the ROI and dividing by the average distance of liver from the coil for the healthy participants. The distance-corrected sodium signal intensities were corrected for the measured relaxation times of the reference solution (T_1_ = 75 ms, T_2_* = 40 ms) using literature values for liver [31] (T_1_ = 34 ms, T_2_* = 17 ms). Estimated sodium concentrations were calculated using the equation

(4) $$TSC=\frac{SI}{m}$$

where *TSC* is the estimated tissue sodium concentration, *SI* is the corrected signal intensity from the liver, and *m* is the slope of the calibration line generated from the corrected signal intensities from the reference vials with the intercept set to zero.

### 2.6. Statistical Analysis

Statistical analyses were performed using R, R Foundation for Statistical Computing, Vienna, Austria [32]. Continuous data were summarized as means and standard deviations, while categorical data were summarized as counts and percentages. Three-way ANOVA was used to test for group differences with group, age, and BMI as the independent variables and the individual MR measures as dependent variables. Fisher’s exact test was used to compare categorical variables. Associations between variables were assessed using Spearman rank-order correlation coefficients. A *p*-value < 0.05 was considered significant for all inference testing. Because this was an exploratory study with a small sample size, no corrections were made for multiple comparisons.

## 3. Results

### 3.1. Study Population

**Figure 1:**
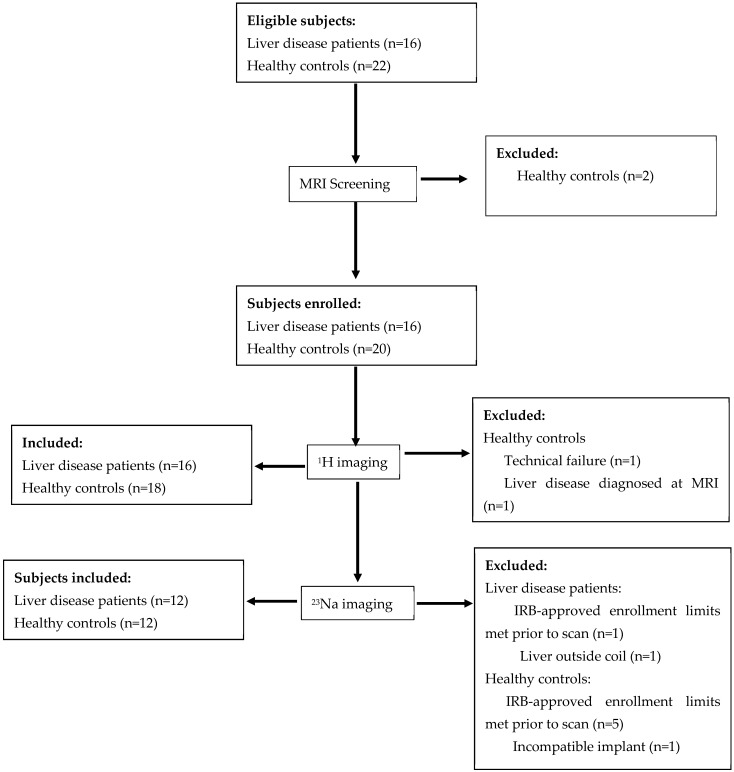
Flow chart showing participant assignment to imaging studies.

A flow chart of participant enrollment is shown in Figure 1.

We identified 40 eligible participants. Two were excluded during pre-study screening and two additional subjects were excluded during the MRI safety screening. Of the remaining 36 participants, we obtained data from 36 participants; one healthy participant was excluded due to technical failure for one or more MRI sequences and one due to liver disease diagnosed at MRI. Thus, the final data set for the APTw imaging study consisted of 18 healthy participants (12 F/6 M) and 16 participants with steatotic liver disease (5 F/11 M). Of the 36 possible participants, 25 were enrolled in the sodium study. Sodium data from one of the liver disease participants was excluded from analysis due to the liver being outside the sensitive volume of the coil, leaving 12 healthy control subjects (9 F/3 M) and 12 participants with steatotic liver disease (4 F/8 M). The patient group ranged in age from 11 to 24 years, and the control group ranged in age from 16 to 25 years. The patient group was younger than the control group (17.5 years vs. 20.9 years, *p* = 0.005) and had a higher BMI (38.2 kg/m^2^ vs. 22.8 kg/m^2^, *p* < 0.001). The two groups were not different by sex (*p* = 0.08). Additional clinical data for the patient cohort are presented in Table 2.

**Figure 2:**
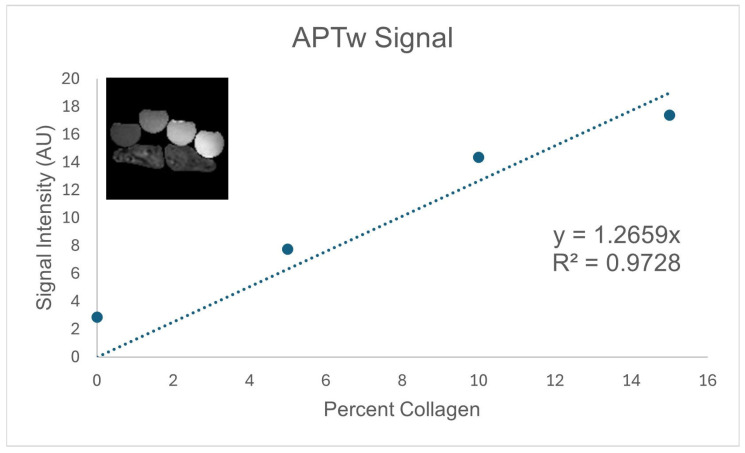
The calibration curve we obtained from the phantom (shown in the inset; collagen phantoms were supported on saline bags with 0%, 5%, 10%, and 15% collagen in the vials from left to right). The intercept was set to zero for this fit. AU: arbitrary units; APTw: amide proton transfer-weighted.

Due to specific absorption rate limits, we were unable to match the RF parameters we used for our murine study. However, the parameters we chose for the saturation transfer experiment provided a linear response with collagen concentration, as shown in Figure 2.

**Figure 3:**
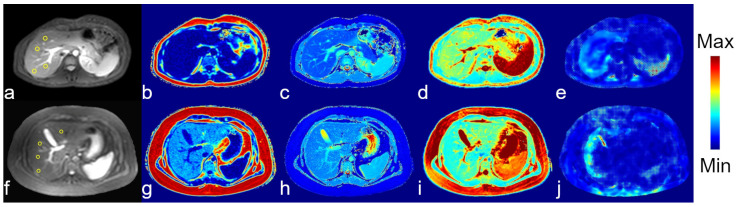
Representative conventional MRI images from a healthy participant (**a**–**e**) and a participant with liver disease (**f**–**j**). Anatomical images (**a**,**f**) from each subject are presented for reference. MRI maps are proton density fat fraction (PDFF: (**b**,**g**)), T_1_ (**c**,**h**), T_2_ (**d**,**i**), liver stiffness (**e**,**j**). The scale for the color bar is 0–100% for the PDFF maps, 0–4200 ms for the T_1_ map, 0–100 ms for the T_2_ map, and 0–10 kPa for the stiffness map. Liver ROIs are shown as yellow circles on the reference images.

**Figure 4:**
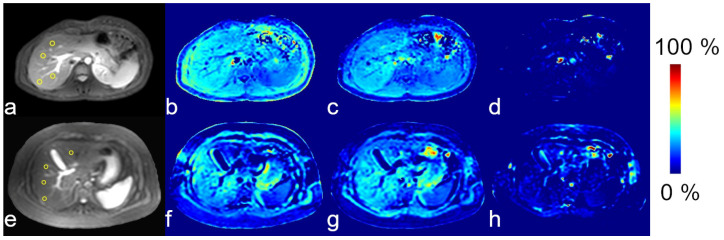
Representative magnetization transfer ratio (MTR) images from a healthy participant (**a**–**d**) and a participant with liver disease (**e**–**h**). Anatomical images (**a**,**e**) from each subject are presented for reference. MTR maps are MTR_−3.5 ppm_ (**b**,**f**), MTR_+3.5 ppm_ (**c**,**g**), and APTw (**d**,**h**). Liver regions-of-interest are shown as yellow circles on the reference images.

**Figure 5:**
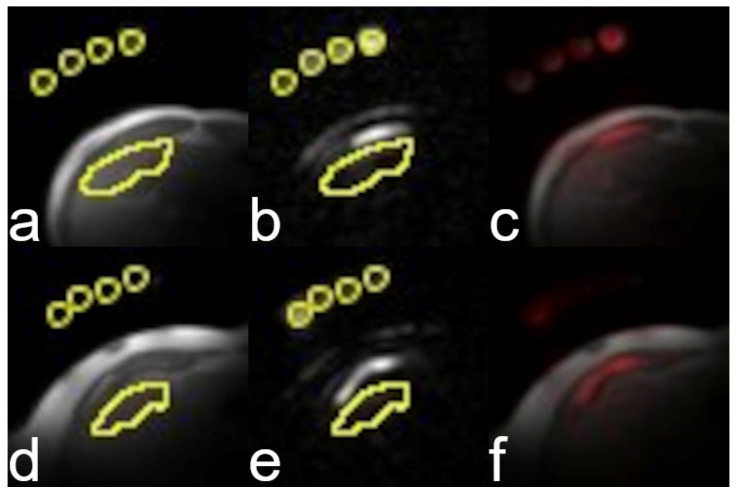
Representative MRI images from a healthy participant (**a**–**c**) and a participant with liver disease (**d**–**f**). Proton anatomical images (**a**,**d**) from each subject are presented for reference. Sodium signal intensity (**b**,**e**) from each subject overlaid in red on the proton image (**c**,**f**) to show sodium signal location. Small circular yellow regions-of-interest (ROIs) are the reference vials. The liver ROI for each subject is also outlined in yellow. The brightest sodium signal comes from the rectus abdominis muscle.

Representative proton images and maps from healthy and patient participants are presented in Figure 3 and Figure 4. Representative sodium images are presented in Figure 5.

### 3.2. Image Quality

Relaxation maps and PDFF maps were acceptable for all participants, defined as having no artifacts in the input images. Areas of acceptable fit for the liver stiffness, as indicated by the lack of hashmarks in the scanner reconstructed images, were found for all but two participants. All MTR maps used to calculate the APTw images were artifact-free, but the resulting APTw maps were noisy. The APTw data from one participant was excluded as an outlier; it was over two standard deviations from the mean. Sodium images for all subjects had signal-to-noise-ratios > 4 in the liver.

### 3.3. Patients vs. Healthy Controls

ANOVA with group, age, and BMI as independent variables showed group differences for liver stiffness (F = 11.2, *p* = 0.003), T_1_ (F = 21.2, *p* < 0.001), and PDFF (F = 19.7, *p* < 0.001). Although APTw did not show group differences (F = 2.0, *p* = 0.2), both MTR_+3.5 ppm_ (F = 12.9, *p* = 0.001) and MTR_−3.5 ppm_ (F = 15.6, *p* < 0.001) did. Liver sodium concentration estimates also differed between groups (F = 12.4, *p* = 0.003). The results of the post-hoc t-tests are given in Table 3.

### 3.4. Associations Between MRI Measurements

Using Spearman’s correlation across all participants, T_1_ correlated with liver stiffness (R= 0.37, *p* = 0.04), PDFF (R = 0.41, *p* = 0.02), MTR_+3.5 ppm_ (R = −0.43, *p*= 0.01), and MTR_−3.5 ppm_ (R = −0.37, *p*= 0.03) but not APTw (R = 0.08, *p* = 0.7). PDFF did not correlate with liver stiffness (R = 0.35, *p* = 0.05) or any of the magnetization transfer parameters but did correlate with T_1_ (R = 0.41, *p* = 0.02) and T_2_ (R = −0.36, *p* = 0.04). T_2_ correlated with MTR_+3.5 ppm_ (R = −0.48, *p*= 0.005) and MTR_−3.5 ppm_ (R = −0.4, *p*= 0.02) but not APTw (R = 0.02, *p* = 0.9). No correlations were found between liver sodium content and any proton measurement.

### 3.5. Associations Between MRI Measurements and Clinical Variables

Across all participants, Spearman’s correlation indicated that BMI was correlated with T_1_ (R = 0.44, *p* = 0.009), PDFF (R = 0.54, *p* = 0.001), MTR_+3.5 ppm_ (R = −0.61, *p* < 0.001), and MTR_−3.5 ppm_ (R = −0.49, *p* = 0.003) but not APTw (R = 0.03, *p* = 0.9) or liver sodium content. Age was correlated with T_1_ (R = −0.40, *p* = 0.02), MTR_+3.5 ppm_ (R = 0.39, *p* = 0.02), and MTR_−3.5 ppm_ (R = 0.45, *p*= 0.007) but not APTw (R = −0.22, *p* = 0.2) or liver sodium content.

Spearman’s correlation in the patient group showed that T_1_ was correlated with ALT (R = 0.72, *p* = 0.002), inflammation (R = 0.62, *p* = 0.01), and NAS score (R = 0.53, *p* = 0.04). T_2_ was correlated with NAS (R = −0.80, *p* < 0.001) and ALT (R = −0.62, *p* = 0.01). PDFF was correlated with NAS (R = 0.75, *p* < 0.001), AST (R = 0.53, *p* = 0.03), and ALT (R = 0.69, *p* = 0.003). T_2_ and PDFF measurements did not correlate with histologic inflammation or fibrosis scores or with the Fib4 score. Liver sodium concentration negatively correlated with the Fib4 score (R = −0.69, *p* = 0.01) but no other clinical measure. Liver stiffness, MTRs, and APTw measurements were not correlated with any laboratory marker of disease (Table 4).

## 4. Discussion

In this study, we planned to investigate the use of APTw and sodium MRI to detect fibrosis in patients with chronic liver disease. However, our patient cohort included only patients with mild steatotic liver disease. Hence, we investigated APTw and sodium imaging in mild steatotic liver disease and compared those measurements to conventional quantitative MRI markers. We also examined the associations between these MRI markers and clinical measures of disease severity, including histologic fibrosis, inflammation, NAS, and Fib4 scores.

The two participant groups differed in age and BMI. The age difference was only 4 years and, thus, probably not clinically relevant. Steatotic liver disease is prevalent in obese subjects [33] who, by definition, have a larger BMI than non-obese subjects. Because we cannot eliminate this confound, we included BMI in our analyses as a covariate. The increased BMI in the liver disease subjects does not negate any findings, particularly given that measures such as PDFF and liver stiffness have proven useful despite also being confounded by BMI.

Like prior studies [34], we found that liver stiffness, PDFF, and T_1_ in the liver were increased in patients compared to healthy controls. We also found that relaxation times correlated with ALT and NAS, while PDFF correlated with those as well as AST. Since fat deposition is a component of NAS, the correlation of PDFF and NAS was expected. We did not anticipate that relaxation times would correlate with either ALT or AST, since those are serum biomarkers that reflect nonspecific liver damage. However, if ALT reflects pathological tissue alterations, then the correlation with T_1_ and T_2_ is reasonable given that these latter measures are known to be sensitive to changes in tissue structure.

The APTw values we found in this study do not agree with our previous murine study, which found APTw values around 8% for mice with fibrosis [22]. Beyond the difference in species and acquisition methods, the mouse livers were highly fibrotic after 16 weeks of treatment, whereas the participants in this study did not have fibrotic liver disease. Hence, we would expect little APTw difference between the healthy control participants and those with liver disease.

The APTw values were negative for both groups because MTR_−3.5 ppm_ was greater than MTR_+3.5 ppm_. Because MTR_−3.5 ppm_ overlaps with the fat resonance, we had expected that the patients with liver disease might show a greater apparent MTR_−3.5 ppm_ due to additional fat suppression, but that was not the case. The MTR_−3.5 ppm_ signal did not correlate with PDFF, which suggests that this signal is independent of fat. Furthermore, our control participants had a greater MTR_−3.5 ppm_ than our liver disease participants, supporting the idea that residual fat is not a confound because the healthy participants had very little fat in their livers. Why the MTR_−3.5 ppm_ signal was greater in the healthy participants remains to be determined.

The APTw maps were noisy due to the similarity of the MTR images that were used to calculate those values. Although MTR values were higher in participants without liver disease than those with liver disease, MTR_±3.5 ppm_ values were similar within groups, leading to small APTw values and increased noise in the difference, as would be expected from a propagation of error analysis.

Both MTRs correlated with measured relaxation times, which agrees with the known effects of relaxation upon chemical exchange saturation transfer measurements. While we hypothesized that the magnetization transfer measurements would correlate with histological measures of fibrosis or clinical indicators thereof, that was not the case. This may be because our final patient cohort consisted of participants with mild steatotic liver disease, who had little or no fibrosis as indicated by both the histologic assessment of fibrosis and the Fib4 Score. Steatosis precedes the development of fibrosis [35], so our patient cohort would not be expected to have significant fibrosis at the disease stage at which they were examined. Alternatively, experimental parameters such as the saturation pulse power or duration may need to be optimized further to improve the detection of fibrosis.

Birchall et al. [36] reported a liver sodium concentration of 41 ± 10 mM in a study of 19 healthy volunteers using a volume coil and volumetric acquisition. Our value of 33 ± 15 mM for our healthy participants is comparable. We hypothesized that the sodium signal would be greater in the patient group due to increased proteoglycan content in fibrotic livers, but the sodium signal was lower in the patient group than in the control group. The lower signal may be due to the distance of the liver from the surface coil in participants with steatotic liver disease, who had much higher BMI values than the healthy control participants. While we attempted to correct this, it cannot be eliminated entirely without B_1_ maps. Sodium levels in the liver did not correlate with any proton MRI measurement.

Liver sodium content was negatively correlated with the Fib4 score. The Fib4 score was derived from the ALT, AST, age, and platelet counts, none of which are markers for or related to liver sodium. The Fib4 score correlation would not survive correction for multiple comparisons and, therefore, may be an artifact. The mechanism we hypothesized to increase sodium in participants with liver disease would have positively, not negatively, correlated with clinical measures of fibrosis. Thus, this finding needs further investigation.

While we did expect to find correlation of liver sodium content with either ALT or AST, based on our previous murine results, the lack of correlation may be due to the fact that ALT and AST levels are not robust measures of non-alcoholic fatty liver disease [37]. Alternatively, it may be due to the low levels of fibrosis in our participants.

Our patient group only included one individual with a histologic fibrosis score greater than 2, which limited our ability to test our hypothesis that either APTw imaging or sodium content would correlate with fibrosis specifically. In general, our patient participants had very mild disease and lacked evidence of advanced fibrosis by either histology or serum biomarkers. All participants had Fib4 scores below 1.45, the cutoff value to exclude advanced fibrosis [24], and only five participants had NAS values greater than 4. Approximately half the patient cohort had ALT levels below 33 IU/L and AST levels below 36 U/L, both within the normal range. None of these clinical markers are direct measures of fibrosis. Sirius Red staining of a biopsy specimen might provide an accurate estimate of liver fibrosis, but even that would only provide information from the sampled region and not the entire liver slice.

Limitations of the study include the aforementioned mild disease, the small sample size, the low spatial resolution of the sodium images, and the inability to collect B_1_ maps to correct the sodium data. Future studies of sodium imaging using surface coils should include B_1_ maps or, minimally, limitations on body habitus to ensure that all subjects’ livers are in approximately the same relative position in the coil. Alternatively, future studies should utilize a volume sodium coil to minimize the effects of B_1_ inhomogeneity on the sodium signal. A study with a wider range of fibrosis markers and/or a wider range of documented fibrosis is needed to confirm the sodium findings. Another limitation of the study was the use of a single slice for both proton and sodium imaging, although other MRI measures of liver disease including MRE, PDFF, and T_1_ are not assessed volumetrically. There is no evidence to date that a volumetric approach is superior to the simpler 2D assessment. However, while the use of a single slice facilitated the correlative data analysis, future work should focus on increasing the volumetric coverage of the liver, both by using volume coils and multi-slice imaging. Sodium is further limited by the need for special hardware to enable its detection that may not be readily available in a clinical setting.

Finally, we did not correct for multiple comparisons in this feasibility study due to the small sample size. A larger sample size with a greater distribution of disease severity is needed to determine the value of these measures. Nevertheless, this work has demonstrated that both methods are feasible in the study population, although neither may be diagnostically useful.

## 5. Conclusions

In conclusion, we found that both APTw imaging and sodium imaging are feasible for liver studies. However, the APTw MRI signal did not differ between participants with and without mild steatotic liver disease, contrary to our hypothesis, and was not associated with any histological marker of disease. Liver sodium content differed between participant groups and was negatively correlated with the Fibrosis-4 score. While both APTw imaging and liver sodium content are biologically plausible markers of fibrosis, larger studies are required to determine if they provide additional value over conventional imaging methods. Sodium imaging is promising but, given that it requires specialized hardware that may not be readily available, its use may be limited compared to conventional methods.

## Figures and Tables

**Table 1:** Research MRI Imaging Parameters.

	T_1_ Mapping	T_2_ Mapping	PDFF/T_2_* Mapping	Elastography	APT	Sodium
**Sequence**	MOLLI (FFE)	TSE	mDIXON Quant	FFE	TSE	FFE
**TR (ms)**	2.42	184	5.2	50	2500	50
**TE (ms)**	1.05	12	0.87	20	46	1.3
**Matrix**	480 × 480	256 × 256	240 × 240	432 × 432	224 × 224	68 × 67
**NSA**	1	1	1	1	1	256
**Breath-hold ^†^**	Y	N	Y	Y	Y	N
**Sequence-Specific Parameters**	Simulated heart rate of 60 beats/minute; 5(3)3 implementation	8 echo times; respiratory triggered	6 echoes	Driver frequency 60 Hz; driver amplitude was set to moderate	16 echoes; ProSet fat suppression; 5 100-ms sinc-gauss RF pulses, 4.2 µT, at offsets of ±3.5 and 23.5 ppm relative to water	Flip angle: 90°

FFE: Fast field echo; mDIXON Quant: modified Dixon sequence; MOLLI: Modified Look-Locker Inversion recovery sequence; NSA: number of signal averages; PDFF: proton density fat fraction; TE: echo time; TR: repetition time; TSE: turbo spin echo; APT: amide proton transfer. ^†^ All indicated sequences used single breathholds except for the APT sequence, which required one breath-hold per frequency offset, or 3 breath-holds in total.

**Table 2:** Patient Participant Clinical Characteristics.

Indicator	Mean (SD)	Range
**Inflammation Score**	1.3 (0.8)	0–3
**Fibrosis Score**	1.3 (0.8)	0–3
**NAS Score**	3.7 (1.4)	2–6
**ALT (U/L)**	98 (99)	7–381
**AST (U/L)**	53 (39)	17–162
**Fib4 Score**	0.37 (0.14)	0.20–0.69

Data are shown as means (standard deviation (SD)). Inflammation and NAS scores were extracted from clinical pathology reports from biopsy specimens. NAS: nonalcoholic fatty liver disease activity score; ALT: alanine transaminase; AST: aspartate aminotransferase; Fib4: Fibrosis-4.

**Table 3:** Derived MRI Parameters by Group.

MRI Parameter	Healthy Controls	Patients	*p* Value *
**T_1_ (ms)**	821 ± 87 (n = 18)	983 ± 111 (n = 16)	<0.001
**T_2_ (ms)**	50 ± 8 (n = 18)	53 ± 16 (n = 16)	0.8
**PDFF (%)**	4.1 ± 1.3 (n = 18)	15 ± 11 (n = 16)	<0.001
**Liver Stiffness (kPa)**	2.1 ± 0.4 (n = 17)	2.8 ± 0.7 (n = 15)	0.004
**APTw (%)**	−5.3 ± 7.9 (n = 17)	−1.9 ± 6.4 (n = 16)	0.2
**MTR_+3.5 ppm_ (%)**	30.2 ± 4.8 (n = 17)	24.3 ± 6.7 (n = 16)	0.008
**MTR_−3.5 ppm_ (%)**	34.7 ± 6.8 (n = 17)	26.2 ± 7.6 (n = 16)	0.002
**TSC (mM)**	33 ± 15 (n = 12)	23 ± 5 (n = 12)	0.046

* From post-hoc t-test. Sample sizes are provided as “n = x” where x is the number of subjects. PDFF: proton density fat fraction; APTw: amide proton transfer-weighted; MTR: magnetization transfer ratio; TSC: tissue sodium concentration.

**Table 4:** Spearman’s Correlation Analysis of Clinical and MR Measurements for Liver Disease Participants.

	Inflammation	Fibrosis Score	NAS	ALT	AST	Fibrosis-4 Score
**Liver Stiffness**	−0.03 (0.9)	0.21 (0.4)	0.33 (0.2)	0.24 (0.4)	0.04 (0.9)	−0.36 (0.2)
**T1**	**0.62 (0.01)**	0.14 (0.6)	**0.53 (0.04)**	**0.72 (0.002)**	0.45 (0.08)	−0.44 (0.09)
**T2**	−0.27 (0.3)	−0.21 (0.4)	**−0.80 (0.0002)**	**−0.62 (0.01)**	−0.49 (0.05)	0.28 (0.3)
**PDFF**	0.40 (0.1)	0.25 (0.3)	**0.75 (0.0008)**	**0.69 (0.003)**	**0.53 (0.03)**	−0.16 (0.5)
**APTw**	−0.34 (0.2)	−0.02 (0.9)	−0.32 (0.2)	−0.19 (0.5)	−0.02 (0.9)	0.26 (0.3)
**MTR_+3.5 ppm_**	−0.02 (0.9)	0.12 (0.6)	0.33 (0.2)	0.18 (0.5)	0.04 (0.9)	−0.04 (0.9)
**MTR_−3.5 ppm_**	0.2 (0.5)	0.06 (0.8)	0.41 (0.1)	0.24 (0.4)	0.07 (0.8)	−0.11 (0.7)
**TSC**	0.11 (0.7)	−0.24 (0.5)	−0.2 (0.5)	−0.39 (0.2)	−0.5 (0.1)	**−0.69 (0.01)**

Values are presented as Spearman’s R (*p*-value). Bold font indicates *p* < 0.05. NAS: nonalcoholic fatty liver disease activity score; ALT: alanine transaminase; AST: aspartate aminotransferase; PDFF: proton density fat fraction; APTw: amide proton transfer-weighted; MTR: magnetization transfer ratio; TSC: tissue sodium concentration.

## Data Availability

The raw data supporting the conclusions of this article will be made available by the authors on request.

## Abbreviations

The following abbreviations are used in this manuscript:

MRI	Magnetic resonance imaging
APTw	Amide proton transfer-weighted
MTR	Magnetization transfer ratio
SI	Signal intensity
RF	Radiofrequency
ROI	Region of interest
PDFF	Proton density fat fraction
ALT	Alanine transaminase
AST	Aspartate aminotransferase
NAS	Nonalcoholic fatty liver disease activity score
Fib4	Fibrosis-4 score
TSC	Tissue sodium content
BMI	Body mass index
